# Education and the Social Evil

**Published:** 1908-02

**Authors:** Alex. W. Stirling

**Affiliations:** Atlanta, Ga.


### Journal-Record of Medicine

Successor to Atlanta Medical and Surgical Journal, Established 1855, and Southern Medical Record, Established 1870.

OWNED BY THE ATLANTA MEDICAL JOURNAL CO.

**Published Monthly.**

*Official Organ Fulton County Medical Society, State Examining Board, Presbyterian Hospital, Etc.*

VOL. IX. FEBRUARY, 1908. No. 11

BERNARD WOLFF, M. D., EDITOR, 319-320 Prudential Bldg.

GEORGE BROWN, M. D., BUSINESS MANAGER, Business Office 1½-5½ S. Broad St.

E. G. BALLENGER, M. D., ASSOCIATE EDITOR AND ASSISTANT BUSINESS MANAGER.

### ORIGINAL COMMUNICATIONS.

### *EDUCATION AND THE SOCIAL EVIL.

BY ALEX W. STIRLING, M. D., C. M., D. P. H., ATLANTA, GA.

Mr. Chairman and Gentlemen:

When I was honored a few days ago by an invitation to open the discussion upon Education relative to the "Social Evil," while regretting the poverty of my acquaintance with the thought of others upon the subject and feeling my inability to lay before the audience with convincing power whatever might be true in what I should have to say, I yet thought that I would merely jot down three or four headings and speak a few words from them. But as I considered the subject, I realized how easy it would be to make myself misunderstood, and decided that I had better commit to paper the result of such ruminations as had taken place in my mind from time to time. This is a subject deserving of much serious consideration. That some action relative to it is greatly needed, and that the medical profession is the best source of such action, was well brought

*Read before the Social Section of the Fulton County Medical Society, Dec. 1907.

out some weeks ago when certain aspects of the so-called "Social Evil" were discussed in this room. Then it was shown that illicit sexual intercourse is at the root of an enormous proportion of the physical ills which curse mankind, not only among those who are themselves guilty, but also among the absolutely innocent, and especially women and children, who frequently never dream the true origin of their distress. To the prostitute, her life is destruction, physical and moral; physical, because it means all kinds of excess and a certainty of venereal disease, with usually but a short life, and apparently, not a very merry one; moral, because it is contrary to conscience, and means contact with, and the contumely of men of depraved tastes and often in a drunken and bestial state of mind. The Social Evil is destructive of the finer sensibilities of the clients of the women for reasons very similar. You will remember how Burns in his "Epistle to a young friend" writes in the hope that he will "better reck the rede, than ever did the advisor:"

"The sacred lowe o' well-placed love,
Luxuriantly indulge it;
But never tempt th' illicit rove,
Tho' naething should divulge it:
I waive the quantum o' the sin,
The hazard of concealing;
But och! it hardens a' within,
And petrifies the feeling!"

It lowers men's regard for women in the mass; it leads to much hypocrisy, and on account of the exceeding frequency of syphilitic, and prehaps yet more on account of gonorrheal infection, it brings incalculable suffering upon many wives and children.

On the other hand, a callous use of the system of prostitution takes the individual so using it out of the arena of one of the most potent struggles in which men engage, and through which they may advance the race towards that perfection of which good men dream, "that far off divine event toward which the whole creation moves." It is, however, only just and wise to separate the man who by force of circumstance, or of temporary loss of control, makes, against his better will, an occasional lapse from the usual upward track in which he walks, and which he immediately strives to regain, from him who smilingly treads the easy path which encourages the prostitute in her unhappy ways and contaminates his own moral being.

If a case has been made out for some form of more energetic private or public interference than has hitherto occurred, the question becomes one of ways and means. The merest glance shows it to bristle with difficulties, and also that in the education of the people generally, and especially of the children, aided by a legislation which will be itself educational, lies the best prospect of success. But as a necessary preliminary, those who would embark in this propaganda must grasp at least something of the task which they are undertaking.

Their ideal is probably absolute continence in every individual until such time as he shall marry, and after that that he shall "love one only and shall cleave to her."

Most of our people believe in monogamy, and probably generally because they have been told it is the only moral state in which married people may live. But when carried back to its origin, it will appear that morality is morality because it is believed to lead to the best physical and mental condition of the people. Measured by this standard, monogamy does appear to be the only moral married state for modern civilization, though something may be said for the polygamy of other times and countries and even for polyandry of such places as Thibet. But the ideals of family life which prevail in the West, and which ought to encourage great virtues among parents and children alike seem to be possible of realization only where there exists in each case a more or less perfect union between one man and one woman. It is in the family that the finest touches are put on character.

Continence and a pure monogamy being then the western ideal, the question becomes: Is such an ideal a possibility for the majority of men? Many, in reply to this question, will answer that such an ideal is preposterous, that prostitution has always existed and will always exist, and that it is indeed essential for the protection of the wives and daughters of the general populace. And yet these same persons would probably be as quick as their neighbors to resent the idea that any of their own people should embark in this necessary and, according to themselves, this saving profession of prostitution. They fail to observe that they are on the horns of an awkward dilemma. Prostitution if necessary can not be immoral, because it stands to reason that no necessary position in life is immoral. If it is not necessary, then sufficient has been said to show that its effects are so destructive physically and morally that it is high time something were done to demolish it. This seems simple enough, but it is essential to look more deeply into the world's history in order to appreciate with accuracy what it means. We must bear in mind that man is still slowly evolving in a kind of upward spiral from forms of life in which conscious moral ideas played no part at all and were even undreamed of, and that each of these imperfect, even sometimes ferocious and bestial states, being a link in the chain of the marvelous, mysterious progress of life, was itself apparently necessary, and therefore not immoral. Our progress toward continence is but one of the lines of growth of our life upward within that Greater Life in which we live and move and have our being. How encumbered that growth must of necessity be, is apparent when we look backwards along the path which we have come. The two most deeply planted instincts among animals are the safeguarding of their own lives and the desire for sexual connection—I can not say for procreation, because that would not be true, the lower animals probably having no idea that the birth of their young has any relationship to the sexual congress. In the great struggle which for hundreds of thousands of years has been progressing between the various branches of animal life, and even between individuals of the same species, only the most fit have survived and many genera have altogether disappeared. The causes of their disappearance were certainly many; but one of the most effcatious was, there can be little doubt, a lack of procreative power. One of the chief physical virtues then among animals was sexual activity. That is equally true of men, and without an exuberant power of procreation, the Anglo-Saxon race, for instance, could never have advanced to its present predominant position in the world.

The greater the sexual energy, then, other things being equal, the better the prospect of survival. Sexual energy has been, therefore, a great virtue from the point of view of the law of survival. This energy among the lower animals has never been curbed by any moral considerations, and how long it was before men themselves were restrained by the gradual evolution of our ideas of sexual morality is apparent by the fact that for thousands of years lineage had to be followed through the mothers, the child not being wise enough to know his own father, and by the number of wives and concubines possessed by even such influential teachers as Solomon and David.

The Greek hetaerai were considered not unworthy companions for the great and noble men of their time, and in some countries of the East even at the present day the prostitutes are the only women who are passably educated. We remember how the crowd melted away and left Jesus alone with the harlot when he invited those without sin to stone her. We know that a Galahad is held up in doubtful admiration even in our own time. We note that religions do not always inculcate continence, and that some have even an opposite tendency.

It is then against an innate, and often an overmastering desire on the part of the male animal to possess the female who attracts his eye, a desire which even at the present, receives much less opposition than that accorded, for instance, to the likewise innate desire to possess his neighbor's goods, and in presence of a public opinion which is distinctly lukewarm, that such as would teach continence have to set their faces. Many will hold such an attempt to be but a waste of time, and the too hopeful will undoubtedly be disappointed. Yet the prophet of evil may well be but a dullard. Such a question is a true test of faith in God and in humanity, and what is hard or impossible to a generation of such savages as we shall be considered a few centuries hence, may be comparatively easy to a race where education will consist, not so much in acquiring a smattering of a number of comparatively trifling subjects, as in drawing out from each individual the highest potentialities of his character, and among whom public opinion will be upon a very different plane from that which our own newspapers form or reflect. But those educators of the present are wise who do not disdain the day of small things, when they remember that the imperfections of the past and of the present may be but essential stages in the ladder of growth, because we "rise on stepping stones of our dead selves to higher things."

In the meantime, the practical question is—what can be done to make easier the path of virtue, more difficult the hitherto easy road of vice? The obvious reply is that it might be simple enough to replace the prevailing dense ignorance on this subject which exists among the great mass of the people, by a knowledge which would do at least something towards lessening physical suffering. Did women know, and it is their right that they should know, what an enormous proportion of their ailments, entailing often pain, operations, sterility and mental anguish is due to disease acquired from the sexual congress, did the public recognize how much blindness, deafness, insanity and many another physical evil, is the result of venereal disease, acquired even many years before, or inherited, something of an upheaval would inevitably ensue. The public should be fully informed in order that it may protect itself upon the physical side.

On the moral side, if it is the opinion of the medical profession that continence is both possible for a properly trained individual and beneficial to the health, and this appears to be the opinion of the most trustworthy of the profession, or even if it were not at first positively beneficial to the physical health of an unaccustomed people, the profession should endorse with no uncertain voice a propaganda against incontinence.

But it is mainly through influence exerted among the young that there is hope for improvement. It is the duty of a child's guardians to teach it on sexual matters in such a manner that, instead of raking, as it were, in the gutters for knowledge, he or she will naturally turn to them for information, and will grow up to look upon this matter with a less excited interest than now results from the mystery purposely exhaled around it.

Fathers should talk to their boys, mothers to their girls, openly upon this subject, so far as they are capable. Many are now entirely incapable of discussing it with proper wisdom, seriousness and restraint, and for these a very carefully written series of pamphlets, which every parent in the country might receive almost cost free, should be prepared, and might well be fathered by the American Medical Association. In it, the method of reproduction among plants and the lower forms of animal life might be used as an easy introduction to that of the higher animals and man—as has of course already often been done. We are always in dread of being, or of appearing to be, indecent; which is a result of what may be our refinement, but is sometimes merely our mock modesty. We have erected certain standards in clothing, any curtailment of which in ordinary life is shocking. And yet under some circumstances, and in response to a, by no means elevated taste, women appear in public in costumes which would not be permissible in private, and which, simply because they are in reality an infraction of our laws of decency, attract our youths and even our elderly gentlemen, and probably do have a demoralizing effect, while they rob the stage of some of its potentially enormous power for good. Decency means a different thing to nearly every country of the world, and our best women when they appear in Western dress in certain Eastern countries, not without cultivated taste, are generally held to be of the harlot class. The narrower the law, the more easily it is overstepped and therefore, in this regard, the more dangerous it is, for a broken law is a law disdained. In like manner, the higher the standard of a man's women friends, the more will he shrink from intimacy with those of a lower type. They are continually educating him for better or for worse. Self control is the great desideratum in temper, habits, mode of thought and of expression. The sense of duty, that "stern daughter of the voice of God" is so little cultivated that one would gladly welcome the imposition of universal military training for boys, and, I had almost said for girls, as well. Whatever trains the character, including games in which combined, rather than individual success is striven for, is of greater value than a bowing acquaintance with Latin and French, though these also may be made excellent means to the same end. I can not endorse the rage for watching others play. I think it encourages uncontrol and—laryngitis,—and is not even restful.

It is also unwise to preach control to children, and at the same time to supply them with beverages, and especially alcohol, which have a stimulating effect upon certain nerve centres and which are generally better asleep.

The educational benefits of wise legislation can hardly be overestimated. It would not be in place here to discuss the effects of the various laws of different countries in relation to prostitution, divorce and kindred subjects; but it may well be remarked that it would appear that the cabinet of no one of the civilized nations has yet estimated the importance of the question in relation to either the health, or the wealth, or the character of the people whom it governs. The rulers of the continent of Europe scarcely seem to have grasped the moral effect of their systems of public brothels; and the women of Great Britain, who rose in arms and refused to "license vice" in their midst, have also failed to perceive the temptations which may assail their sons and brothers who, perhaps after dining not wisely but too well, might in better regulated streets have wended their homeward way without hurt.

The law of public opinion is nearly as powerful as that of the bench, and it has condemned erring women with a bitterness withheld from men, perhaps, to be just to it, because it has felt line from virtue and therefore less worthy of condemnation. In that it is comparatively easy and cheap for a man to cross the line from virtue and therefore less worthy of condemnation. In this, there certainly seems to be some truth, especially if we agree with the poet, who, addressing the censorious and rigidly righteous among the sisterhood, suggested that their own virtue might perhaps be due to their being "aiblins nae temptation," and begged them to

"Think, when their castigated pulse
Gies now and then a wallop,
What ragings may his veins convulse
That still, eternal gallop."

This man knew other men, and he knew women, alas! too much, and he was generous to them all. So he wrote:

"Then gently scan your brother man,
More gently sister woman;
Though some may gang akennin' wrang,
To turn aside is human.
One point must still be greatly dark,
The moving why they do it;
And just as lamely can ye mark
How far perhaps they rue it.
Who made the heart, 'tis He alone
Decidedly, can try us;
He knows each chord, its various tone,
Each string, its various bias.
Then at the balance, let's be mute,
We never can adjust it;
What's done we partly may compute,
But know not what's resisted."

Perhaps a more censorious spirit than that of the poet would prove more stimulating to virtue, but it seems to me that while the ideals to be aimed at cannot be too high, the facts of human evolution must never be lost sight of if education is to be upon natural lines and is to exhibit that insight into the potentialities of the pupil, which is a necessity for success. When all is said and done there can be no royal road to purity. It is not chiefly by teaching children what to avoid that they can be educated. It is mainly by instilling into their minds the beauty of following the right for its own sake, by cultivating in them a positive ambition, by encouraging them so to fill their minds with lofty and soaring enthusiasms, their religion, in fact, that the low and groveling can find no place therein; it is only thus that assurance can be had that our children will pass clean through the ever-menacing contaminations of life.

